# Correction to “Circ‐PDZD8 promotes cell growth and glutamine metabolism in non‐small cell lung cancer by enriching LARP1 via sequestering miR‐330‐5p”

**DOI:** 10.1111/1759-7714.15148

**Published:** 2023-11-08

Zhu X, Du T, Chen X, Hu P. Circ‐PDZD8 promotes cell growth and glutamine metabolism in non‐small cell lung cancer by enriching LARP1 via sequestering miR‐330‐5p. *Thorac Cancer*. 2023;14(22):2187‐2197. https://doi.org/10.1111/1759-7714.15006

The authors would like to add the below information to the article:

Xiaopeng Zhu and Tianxing Du contributed equally to this work.

We apologize for this error.

